# Supplementary figures and images for: Oral Microbiota from Periodontitis Promote Oral Squamous Cell Carcinoma Development via γδ T Cell Activation
Source: mSystems. 2022 Aug 24;7(5):e00469-22. doi: 10.1128/msystems.00469-22 (PMC9600543; doi:10.1128/msystems.00469-22)

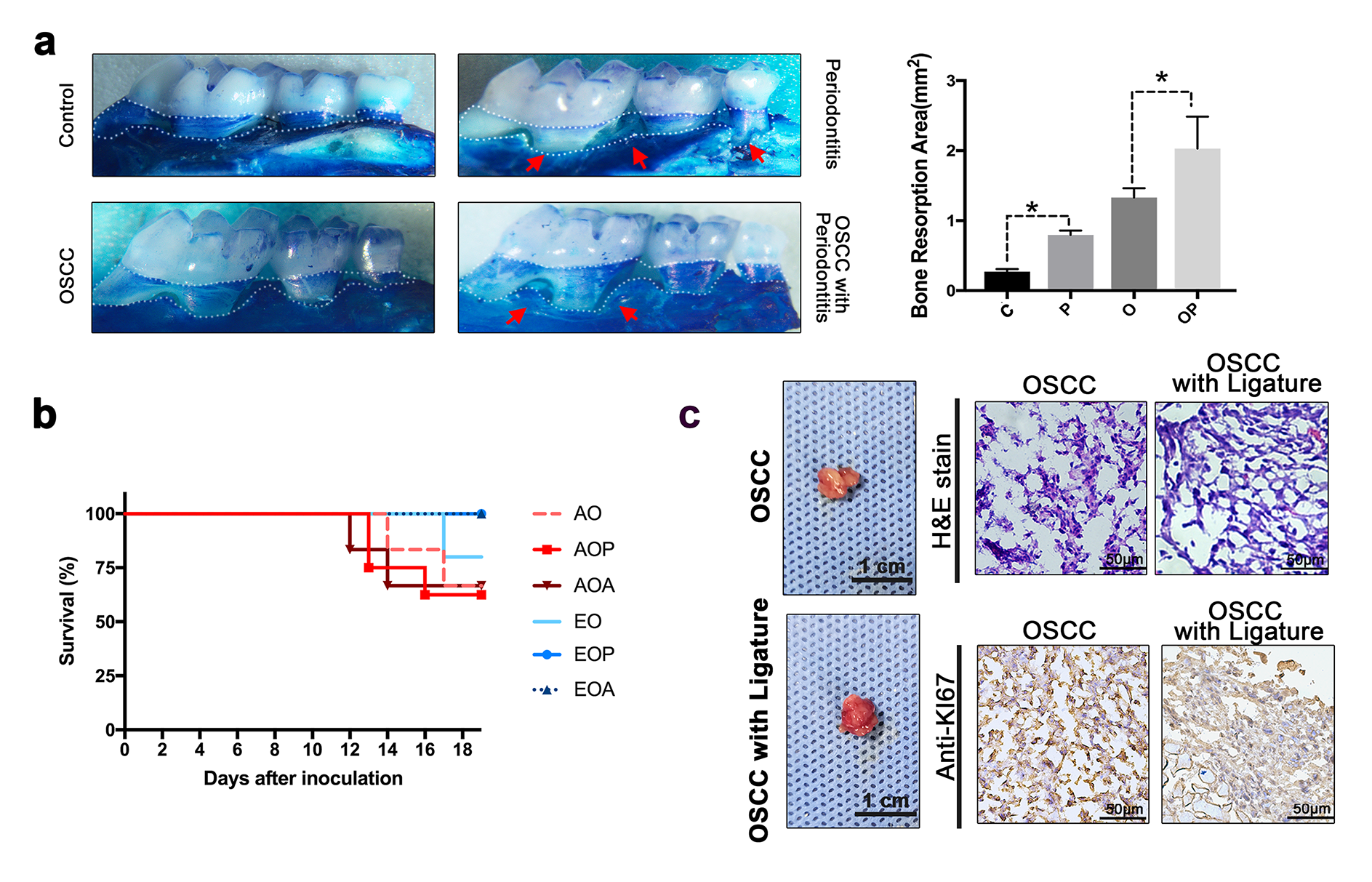

Supplement: FIG S1 [file msystems.00469-22-s0001.tif]

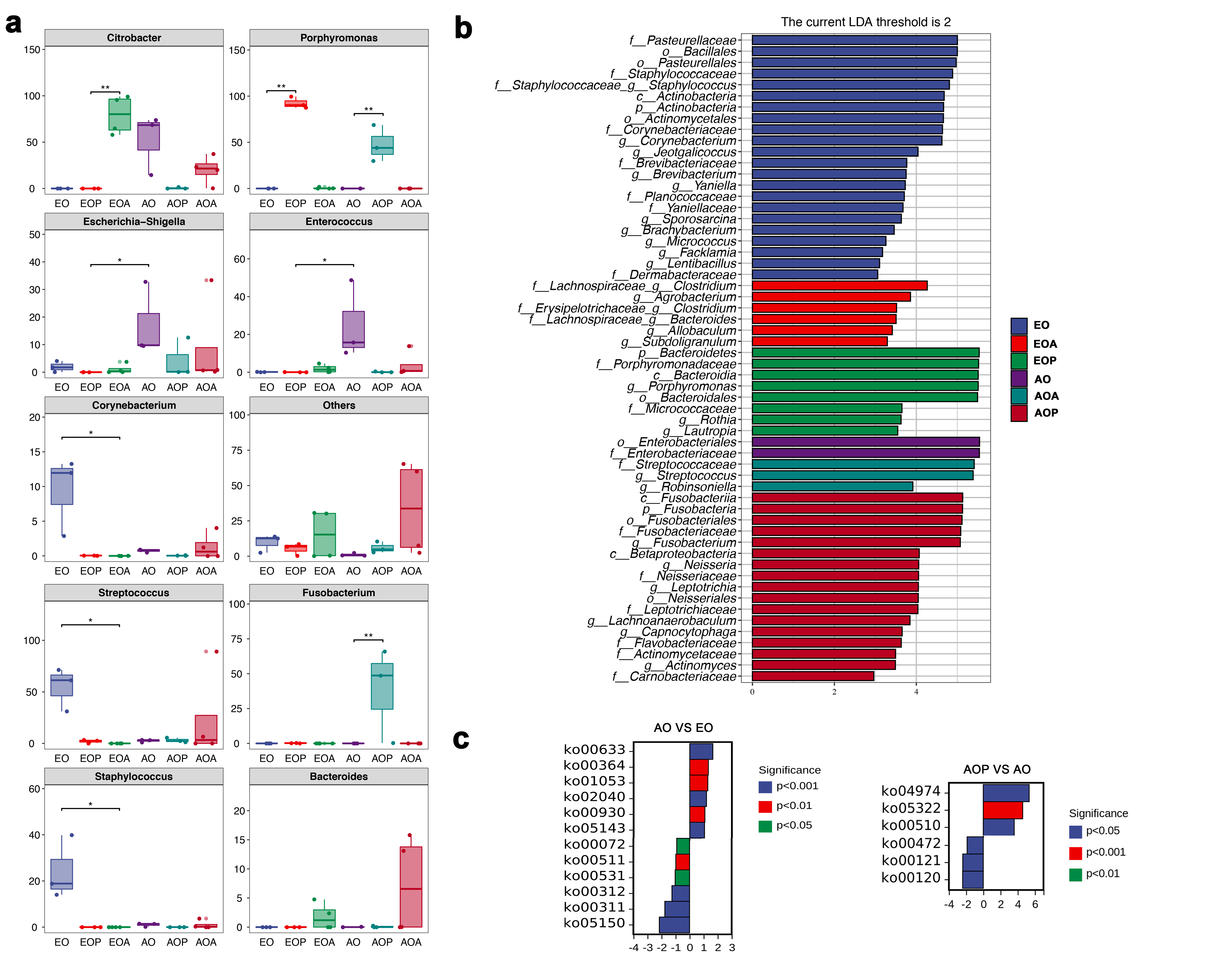

Supplement: FIG S2 [file msystems.00469-22-s0002.tif]

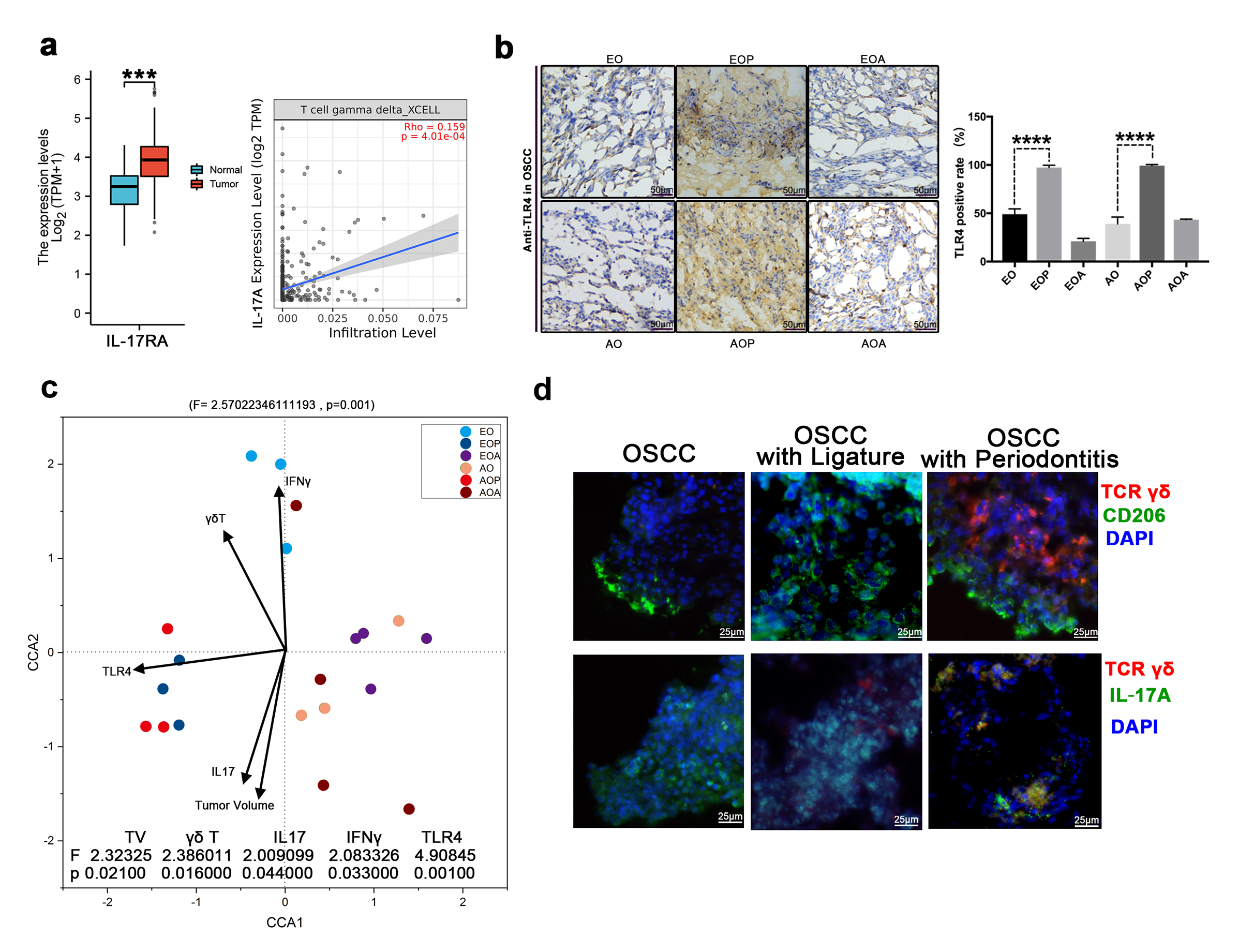

Supplement: FIG S3 [file msystems.00469-22-s0003.tif]

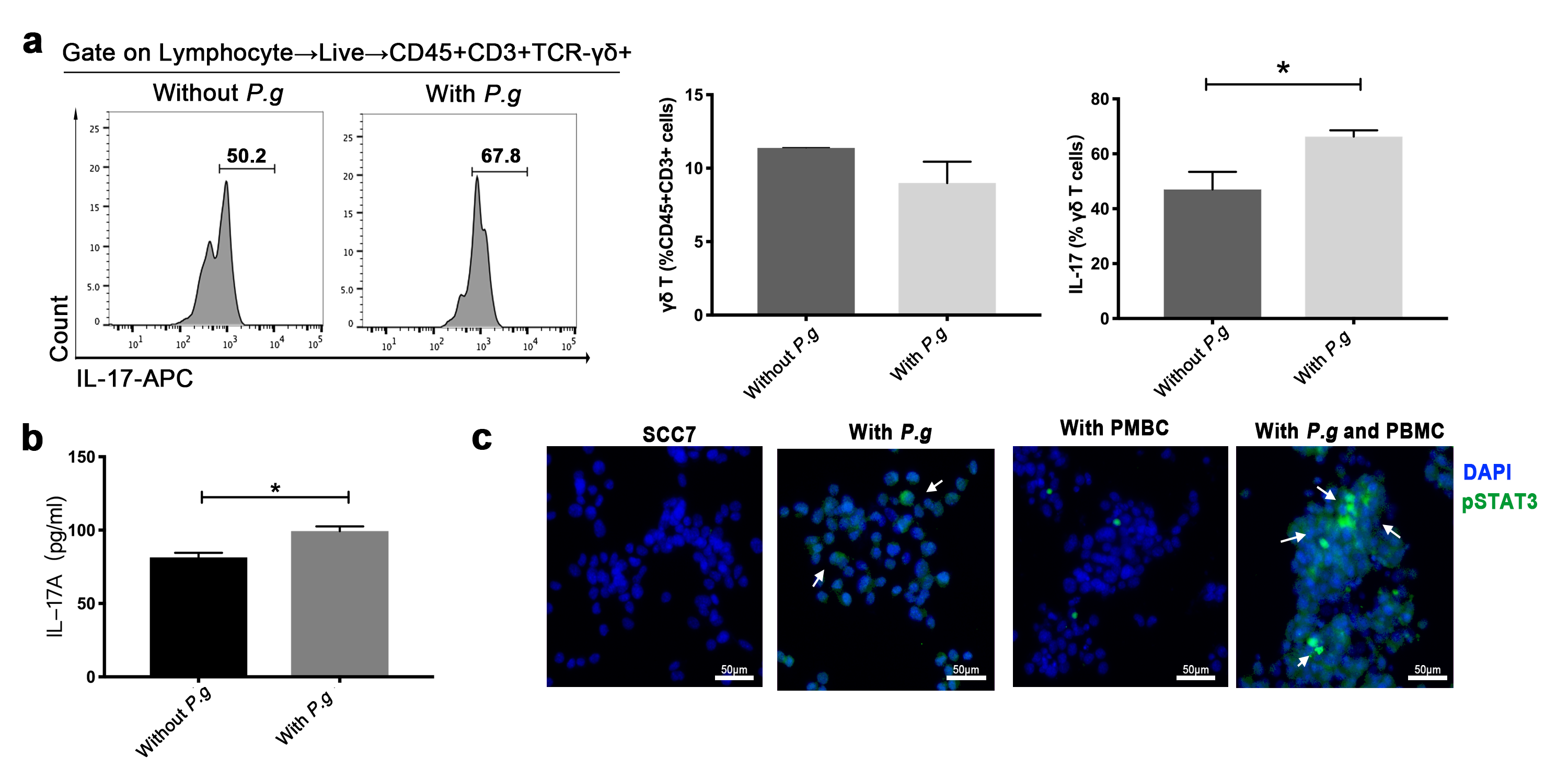

Supplement: FIG S4 [file msystems.00469-22-s0004.tif]

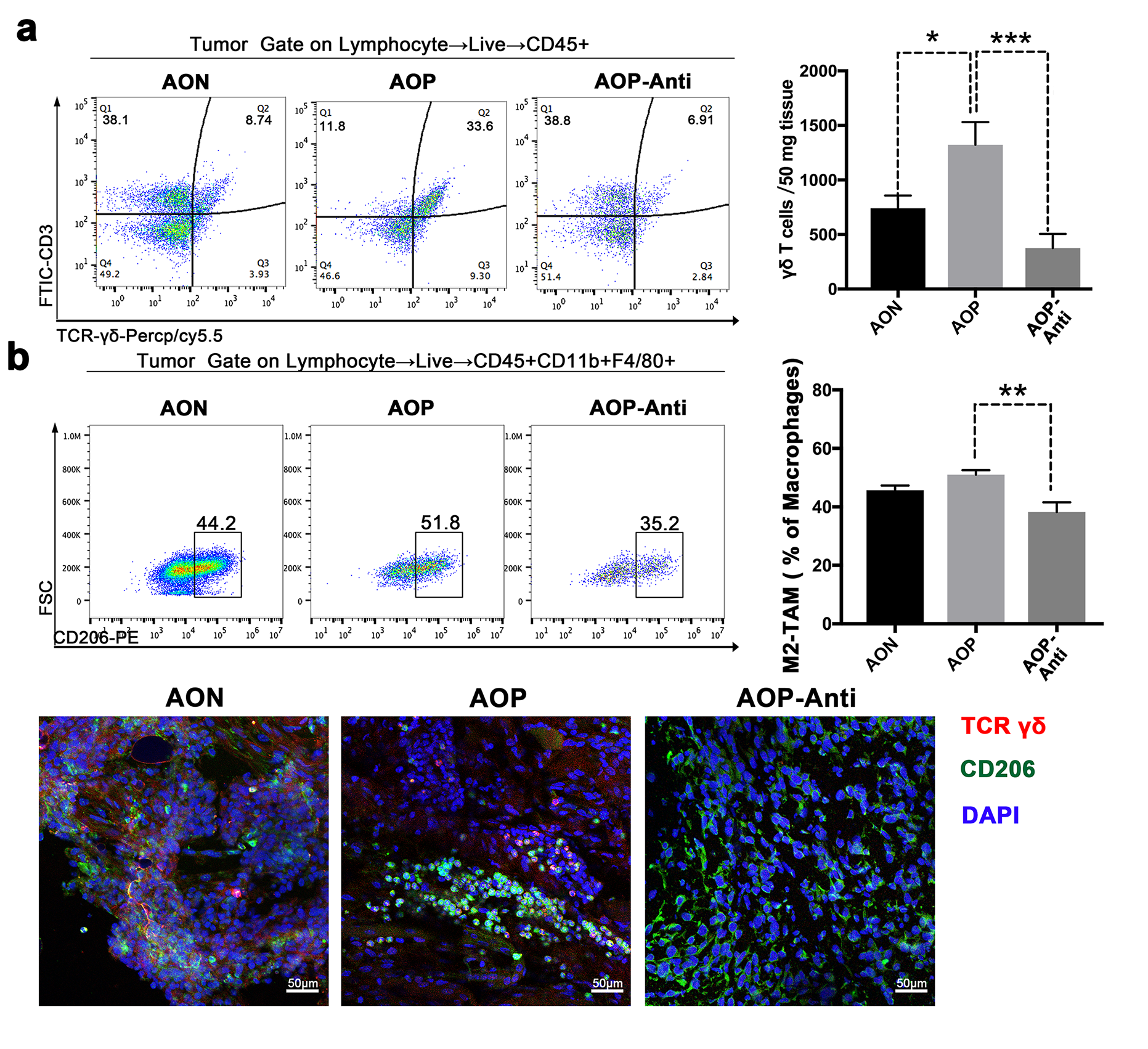

Supplement: FIG S5 [file msystems.00469-22-s0005.tif]
